# Tobacco smoke incursion into private residences in Israel: a cross-sectional study examining public perceptions of private rights and support for governmental policies

**DOI:** 10.1186/s13584-023-00573-w

**Published:** 2023-07-21

**Authors:** Noa Theitler, Vaughan W. Rees, Maya Peled-Raz, Michal Bitan, Laura J. Rosen

**Affiliations:** 1 Department of Health Promotion, School of Public Health, Faculty of Medicine, Tel Aviv University, POB 39040, 69978 Ramat Aviv, Israel; 2 Israel Ministry of Education, Tel Aviv, Israel; 3 Department of Social and Behavioral Sciences, Harvard T.H. Chan School of Public Health, Boston, MA USA; 4 School of Public Health, University of Haifa, Haifa, Israel; 5 School of Computer Science, College of Management, 7502501 Rishon LeZion, Israel

**Keywords:** Tobacco Smoke Exposure (TSE), Secondhand smoke (SHS), Tobacco Smoke Incursion (TSI), Attitudes, Smoke-free housing, Multi-unit housing, Neighbor smoking, Residential exposure, Support for policy, Secondhand Smoke (SHS)

## Abstract

**Background:**

Tobacco smoke incursion (TSI) into private residences is a widespread problem in many countries. We sought to assess the prevalence of self-reported TSI and public attitudes about TSI in Israel, a country with a relatively high smoking prevalence and high population density.

**Methods:**

We conducted a random digit dial survey among residents in Israel (N = 285) in 2017, which examined the frequency, source, correlates of, and attitudes towards TSI and potential regulatory options. The cooperation rate was 63.9%.

**Results:**

Among respondents, 44.7% reported ever experiencing home TSI, with higher exposure among residents of multi-unit housing (MUH) (MUH versus private homes: aOR (Adjusted Odds Ratio): 3.60, CI (Confidence Interval): [1.96, 6.58], *p* < .001). Most respondents (69.8%), including nearly half of smokers, prioritized the right of individuals to breath smoke-free air in their apartments over the right of smokers to smoke in their apartments. Women and non-smokers were more likely to support the right to breathe smoke-free air (Women versus men: aOR: 2.77 CI: [1.48, 5.16], *p* = .001; Nonsmokers versus smokers: aOR: 3.21 CI [1.59, 6.48], *p* = .001). However, only about a quarter (24.8%) of respondents who ever experienced TSI raised the issue with the neighbor who smoked, the neighbor's landlord, or the building committee. The vast majority (85.2%) of all respondents, including three-quarters of smokers, supported smoke-free legislation for multi-unit housing (MUH), with those ever-exposed to TSI and non-smokers more likely to support legislation (ever-exposed versus never-exposed aOR = 2.99, CI [1.28, 6.97], *p* = 0.011; nonsmokers versus smokers aOR = 3.00, CI [1.28, 7.01], *p* = 0.011).

**Conclusions:**

Among study participants, tobacco smoke incursion was a common, yet unwelcome experience. Most respondents believed that the right to breathe smoke-free air in one's apartment superseded that of neighbors to smoke anywhere in their home, and most supported legislation to prevent TSI. Though further study is needed to understand better TSI and effective methods for its prevention, our findings suggest that policy interventions, including legal action at the level of the Supreme Court and/or the Knesset, are needed. Regulation, policy initiatives and campaigns to denormalize smoking in proximity to other people and private residences globally could reduce the scope of this widespread problem, protect individuals from home TSI, and improve population health.

## Background

Laws governing smoking in public indoor and outdoor spaces have become common in Western societies, due to the known harms of secondhand smoke (SHS) exposure [1, 2]. Harms include early death and disease among nonsmoking adults and children. Children are particularly susceptible to SHS: child exposure increases risk of sudden infant death syndrome, respiratory and ear infections, more severe asthma, and retarded lung development [3]. Indeed, protection from secondhand smoke exposure is a key article under the World Health Organization (WHO)'s Framework Convention for Tobacco Control (FCTC) [4], and is one of 6 pillars of WHO's MPOWER policy implementation strategy [5]. Remarkable progress has been made in smoke-free laws over the past two decades. Population levels of SHS exposure have decreased dramatically in countries with strong smoke-free policies. For example, child exposure to SHS, measured using objective biomarkers of exposure, fell by 90% in the UK between 1998 and 2018, with an acceleration in adoption of smoke-free homes observed following the 2007 ban on smoking in public places [6]. Multiple studies have demonstrated decreases in second hand smoke (SHS) exposure in jurisdictions that have adopted smoke-free legislation, including Scotland, Ireland, and U.S. states Michigan and New York. Despite these gains in protection from SHS exposure, an estimated 40% of children and a third of nonsmoking adults remain unprotected from the harmful effects of SHS exposure [7]. This gap in protection may be attributable not only to failures to uniformly adopt or enforce existing smoke-free policies globally, but also to failures to advance new policies that would protect people from SHS in a setting where significant exposure occurs: private homes.

Despite the demonstrated benefits of smoke-free laws in workplaces and other public venues, a substantial proportion of the public continues to be exposed to SHS in residential environments [8]. Private areas, including homes, are less feasible to regulate via legislation, though courts have directed parents or caregivers to provide a smoke-free environment as part of child custody arrangements [9]. Home smoking attitudes and behaviors vary widely around the globe: in some societies, smoking in the home is frowned upon, while in others, offering a cigarette to a (male) guest is considered common courtesy [10, 11].

Yet, even eliminating smoking within the home does not necessarily protect residents from SHS exposure, because smoke may penetrate the home from outside. Tobacco smoke incursion (TSI) can occur when tobacco smoke enters a private home through doors, windows, ventilation systems or other openings [12]. The risk is greater with closer proximity to other dwelling units, as may occur in multi-unit housing (MUH) and/or in areas where buildings are close together. Children in nonsmoking residences living in MUH in the U.S. have been shown to have higher exposure levels than those living in private homes [13], suggesting that TSI may be an important exposure source. Neighbor smoking may be related to disease outcomes: a study of over 60,000 adolescents in Hong Kong found an association between exposure to neighbor smoking and higher rates of respiratory symptoms [14], and a study of 17,000 children in Korea found that smoke penetration into homes was associated with greater incidence of wheeze, rhinitis, and eczema [15]. In Israel, the combination of high smoking rates (20.1%) [16] and a high proportion of residents who live in multi-unit housing (74%) [17–19], suggests that TSI may present a serious and pervasive health risk to a substantial proportion of the Israeli population.

The aim of this study was to assess the prevalence of TSI among Israelis, and gauge public perceptions regarding the rights of smokers to smoke in their homes relative to the rights of neighbors to breath smoke-free air in their homes. We also assessed support for policies to protect residents from TSI, the prevalence of tobacco smoke incursion, and attitudes regarding TSI.

## Methods

### Study design and sampling strategy

We conducted a cross-sectional, random digit dial survey of mobile phone users in the Hebrew-speaking adult population of Israel (ages 18+), between August-December 2017. Use of cellphones is high in Israel: in 2017, the per-person average was 1.25 [20]. Phone numbers were generated using the Excel function for creating random numbers with 10 digits using existing Israeli phone codes (0502000000–0589999999). Those who were unwilling to respond by phone, or for whom it was difficult to schedule a time, were offered an option of completing a web-based survey. A target sample size of N = 250 was calculated with the goal of achieving 5% precision on a conservative estimate of TSI of 20% of the population, using the WinPepi program [21].

### Survey measures (self-report)

a) *Socio-demographic variables* included: gender (male/female), age, religion (Jewish, Muslim, Christian, Druze, other), religiosity (secular, traditional, religious, Ultra-Orthodox, other), educational status (<= 12 years, > 12 years), type of dwelling (single-family, two-family home, or MUH with more than 2 units) and financial status (“How would you define your financial status” categorized into <=average, above average).b) *Smoking and home smoking practices*: Participants were asked “Do you smoke?” with possible responses: Yes, daily or almost daily; Yes, sometimes; Yes, specified times (Social events, army service, stressful periods); Former smoker; Tried but I was never a regular smoker; Never smoker.

Individuals who responded that they smoked “sometimes” or more frequently were categorized as smokers. Those who smoked only at specified times, those who didn’t smoke at all, and former smokers were categorized as non-smokers. Respondents were asked whether they lived with a smoker (regardless of whether they smoked in the home) (yes/no), whether anyone smoked in the home, near a window or on a porch (yes/no), and, if so, how often (daily or nearly daily, sometimes, rarely, never), and whether a neighbor had ever complained (yes/no).c) *Smoke incursion and strategies used to prevent or reduce incursion*: We asked about whether the respondent had ever experienced tobacco smoke incursion in the home (Yes/No) with the question, “Have you ever seen, felt, or smelled tobacco smoke which penetrates your home as a results of someone smoking in a nearby apartment, in the hallway, or in the area of your building? “ Among those reporting ever-incursion, we asked how often it had occurred in the past month (More than once a day, daily or almost daily, several times per week, once a week, 1-3 times per month, not at all). We recorded the season of the response (summer/fall/winter/spring). We asked where smokers were at the time of smoke incursion (in an apartment above, on the same floor, below, from an adjacent building, in the stairway, in the building entrance, in the common garden of the building), and by what venue the smoke entered the home (e.g. window, entrance, porch). We asked whether respondents took any mitigation strategies, including closing windows or doors, or approaching the smoker, landlord or building committee to find ways to reduce the incursion.d) *Risk perceptions and attitudes towards TSI*: Among those who experienced TSI in the past month, we asked about to the extent to which respondents thought that tobacco smoke incursion into their homes was a health risk (4-point scale: Very harmful to Not harmful). This was categorized into a binary variable (Very harmful or harmful versus not so damaging or not damaging at all). We also asked those respondents the extent to which they were troubled by the smoke incursion (6-point scale: I moved/sued my neighbor; I plan to move/sue my neighbor; it disturbs me a lot; it disturbs me; it doesn’t disturb me that much; it doesn’t disturb me at all.) This variable was categorized into a binary variable (disturbed/not disturbed, where “not disturbed” included the categories doesn’t bother me much / doesn’t bother me at all.e) *Responsibility and rights*: We asked who was responsible for prevention of TSI to other residential units (i.e. the government / the smoker/ the person into whose home the smoke infiltrates / the owner of the smoker's apartment / the owner of the apartment which is infiltrated/ the building committee/ no one / other). We asked which right took precedence: the right to smoke anyplace in one’s home even it bothers the neighbors, or the right of neighbors to breathe clean air in their own apartments.f) *Support for regulation*: We asked about support(yes/no) for smoke-free policies in multi-unit dwellings, as follows: whether respondents would support a law forbidding smoking in common apartment building areas (e.g. stairways, entrances, shared gardens) and/or a law forbidding smoking in private areas of apartments (porches, apartment interiors). We created binary variables to reflect support for any smoking policy, support for policy in specific common areas, and support for policy in specific private areas.

### Weighting

Population level distributions were obtained from the 2017 Social Survey conducted by the Central Bureau of Statistics (CBS) of Israel, using the Table Generator function on the website [22]. Because the CBS data included persons aged 20+, and our data included persons 18+, we adjusted the weights from numbers obtained from the CBS by adding 10% to the raw numbers in the youngest age group, i.e., 18-39. We note that our question on current smoking was slightly different from the CBS question. We asked about cigarettes, cigars, pipes, nargila (hookah), electronic cigarettes (vaping) and IQOS (a heated tobacco device). The CBS asked about cigarettes, cigars, pipes, and nargila, but not electronic cigarettes or IQOS. This would be a problem if individuals used only electronic cigarettes or IQOS. However, use of electronic cigarettes in Israel is low. Data from an internet panel survey which we conducted in 2020 showed that just 6 of 406 respondents used e-cigarettes or IQOS, and all of those were current smokers of combustible cigarettes or nargila [23]. Eight cell weights were calculated, for each combination of population group (Jews & Others/ Arabs), sex (female/male), and current smoking status (yes/no), by dividing the cell percent from the CBS data by the cell percent from the data in the current survey.

### Response rates

We calculated the response rates using the calculator provide by the American Association of Political Opinion Research (AAPOR) [24].

### Statistical analyses

We describe the distributions of sociodemographic variables using unweighted data. All other analyses are presented using weighted data.

We examined: (1) smoking behavior of the respondent and family members and neighbor complaints; (2) tobacco smoke incursion; (3) risk perceptions; (4) responsibility and rights; and (5) support for legislation among smokers versus nonsmokers, using Chi-squared tests for categorical variables or Wilcoxon Mann Whitney tests for ordered variables.

We used multivariable logistic regression to examine the relationship between potential predictors and three outcome variables. The outcomes variables were: ever experienced TSI (yes/no); rights of smokers versus rights of neighboring residents; and support for legislation for protection from TSI in MUH (yes/no). The predictor variables used in models of all outcome variables were: smoking status (current smoker yes/no); building type (MUH, cottage/dual family; private); educational level (≤ 12 years/ 12 years + ); family income (≤ Average, > Average), age of respondent (continuous), sex of respondent (male/female), and whether at least one child under the age of 18 lived in the home (yes/no). For the models evaluating smoker versus neighbor rights and policy support, we also included ever exposure to TSI (yes/no) as a potential predictor. Full models (that is, all specified variables were included) were used in all cases. Statistics were calculated using SPSS Version 28 with a 2-tailed significance level of 0.05 and 95% confidence limits.

## Results

### Response rate

**Fig. 1:**
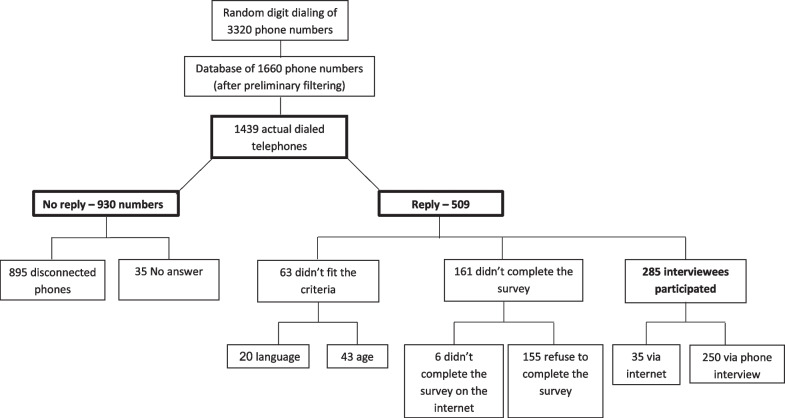
Flow chart of recruitment and participation

We attempted to contact 1439 phone numbers from our list of 3320 computer-generated numbers. There was no answer from 64.6% (930/1439) of the numbers, mostly due to numbers which did either not exist, were disconnected, or turned off. Among the 509 phones numbers where potential respondents were reached, 12.4% (63/509) were not eligible due to age or language. Among those 446 telephones which were answered and for which the potential respondent was eligible, 285 agreed to participate. According to the AAPOR calculator, the cooperation rate was 63.9%, Response rates 1 and 2 were 59.3%, and Response rates 3 and 4 were 62.3% [24] See Figure 1.

### Participants

In total, 285 respondents participated in the study during 2017. Of those, 250 answered the phone survey and 35 answered via the internet. Women comprised 53.3% (N = 152) of respondents as compared with 52.2% in the population as a whole. The average age was 40.3 (N = 273, SD 16.8), 87.2% were Jews (as opposed to 76% in the population), 53.9% were secular (population: 56.6%), 66.4% had greater than high school education (population: 52.6%). The majority (87.4%) lived in urban areas (population: 91.2%), and more than half (54.7%) lived in multi-unit apartment buildings, 31.7% in single family (private) homes and 13.7% in dual (two) family homes. Overall, 5.6% answered in August (summer), 2.4% in October (fall), 52.3% in November (fall) and 39.6% in December (winter). About half of the respondents (50.2%) reported having a child under the age of 18 living with them. The number of unweighted observations available for each analysis are presented in Table 1, which describes demographic data by smoking status.

**Table 1:** Demographic variables by smoking status (Unweighted data)

Question Number of participants available for analysis	Possible answers	Nonsmokers % N	Smokers % N	All % N	*p* values
Building type N = 278	Multi-unit building	56.9% 115	48.7% 37	54.7% 152	0.291
	Two-family cottage	11.9% 24	18.4% 14	13.7% 38	
	Private house	31.2% 63	32.9% 25	31.7% 88	
Educational status N = 283	12 years or under	35.6% 73	28.2% 22	33.6% 95	0.239
	Above 12 years	64.4% 132	71.8% 56	66.4% 188	
Economic status N = 276	Average and under	72.0% 144	72.4% 55	72.1% 199	0.951
	Above average	28.0% 56	27.6% 21	27.9% 77	
Children at home N = 281	No	50.2% 102	48.7% 38	49.8% 140	0.819
	Yes	49.8% 101	51.3% 40	50.2% 141	
Sex N = 285	Male	46.6% 96	46.8% 37	46.7% 133	0.972
	Female	53.4% 110	53.2% 42	53.3% 152	
Religion N = 281	Jewish	87.3% 179	86.8% 66	87.2% 245	0.916
	Not Jewish	12.7% 26	13.2% 10	12.8% 36	
Religiosity N = 280	Secular	54.5% 110	52.6% 41	53.9% 151	0.841
	Not secular	45.5% 92	47.4% 37	46.1% 129	
Urban_Rural N = 261	Rural	13.8% 26	9.6% 7	12.6% 33	0.355
	Urban	86.2% 162	90.4% 66	87.4% 228	
Season N = 285	Summer	3.4% 7	11.4% 9	5.6% 16	0.025
	Autumn	57.3% 118	48.1% 38	54.7% 156	
	Winter	39.3% 81	40.5% 32	39.6% 113	
Age N = 273	Mean, Std, N	42.5 (17.7) 197	34.5 (12.8) 76	40.3 (16.8) 273	< 0.001

### Smoking behavior and neighbor complaints

Among respondents, 23.5% reported that they were current smokers. Smoke-free homes were reported by half (50.5%) of respondents (Smokers: 22.1%, Nonsmokers: 59.4%; p<.0001). Among those respondents who reported that smoking occurs in their home, near a window or on a porch, just 7.1% reported that a neighbor had ever complained about smoking coming from their home (Smokers:13.5%, Nonsmokers: 3.4%, *p* = 0.024).

### Smoke incursion and strategies used to prevent or reduce TSI

Overall, 44.7% reported ever experiencing TSI, 46.5% reported never experiencing TSI, and 8.8% could not recall. TSI entered primarily through windows (69.3%) and/or porches (51%), particularly following smoking by one of the neighbors. 37.9% reported that the smoker was located on a floor below, 17.9% reported that the smoker was on the same floor, 15.8% reported that the smoke came from the floor above, and 16.5% reported that the smoke came from an adjacent building. Other sources of TSI included smoking in stairwells (23.0%), in building entrances (22.7%), in common garden areas (21.9%), and from the street (17.3%). About a third of respondents (34.7%) reported that the incursion was caused by more than one source.

Nearly three quarters of ever-exposed respondents (70.1%) took steps to reduce the exposure in their homes. The most common approach was closing windows (46.7%). Only a quarter of all those ever exposed (24.8%) reported that they ever tried to speak with the smoking neighbor, the neighbor’s landlord, or the building committee to reduce TSI. The reasons for not approaching reasons were: a) the respondent felt that they didn’t have the right to tell the smoker not to smoke (39.7%), (b) the smoke didn’t bother them that much (32.5%), (c) the respondent felt that a conversation with a smoker wouldn’t help (24.1), (d) the respondent didn’t know who was smoking (10.7%), or (e) the respondent feared entering into a conflict with the smoker (9.5%). (Note: multiple answers were permitted, so the percentages do not sum to 100.) Among those who approached the smoker, just 11% reported that it helped them to reduce exposure substantially, 53.1% reported that it helped somewhat and 19.3% said it hadn’t helped at all. A major argument was reported by 16.5% of respondents who approached their neighbor. See Table 2.

**Table 2:** Tobacco use behavior, incursion, risk perceptions and attitudes by smoking status

Topic	Question Number of participants available for analysis (N_U_: Number Unweighted)	Possible answers	Nonsmokers (%)	Smokers (%)	All (%)	*p* value
Tobacco use behavior and neighbor complaints	Q1. All respondents: Do you smoke?)Including: Cigarettes, cigars, pipes, nargila, e-cigarettes, IQOS, (Not including cannabis)) (N_U_ = 285)	Yes, daily or almost daily	NR	NR	21.4	
		Yes, sometimes			2.1	
		Yes, specified times (Social events, army service, stressful periods)			2.5	
		Former smoker			14.7	
		Tried but I was never a regular smoker			12.6	
		Never smoker			46.7	
	Q1. Among respondents who smoke or smoked in the past: What do you smoke or did you smoke in the past?) (Nu = 161) *Can check more than one answer	Boxed cigarettes	88.1	74.6	82.1	< 0.001
		Roll your own cigarettes	14.3	47.8	19.9	
		Cigars	1.2	3.0	2.0	
		Nargila	16.7	16.4	16.6	
		Pipe	1.2	1.5	1.3	
		Electronic cigarettes	0.0	6.0	2.6	
		IQOS	0.0	1.7	0.7	
	Q2. All respondents: Do you live with a smoker (not including yourself), (regardless of whether s/he smokes in the home)? (N_U_ = 284)	Yes	26.3	61.2	34.5	< 0.001
		No	73.7	38.8	65.5	
	Q3. All respondents: Does anyone in your house smoke in the home, near a window or on a porch (not including garden, stairwell, or parking)? (N_U_ = 284)	Yes, daily or almost daily	13.8	52.9	23.2	< 0.001
		Yes, sometimes	8.8	13.2	9.8	
		Yes, but rarely	18.0	11.8	16.5	
		No, never	59.4	22.1	50.5	
	Q3. Among respondents where smoking occurs in the home, near a window or on a porch: Where does smoking take place? (N_U_ 144) *Can check more than one answer	On a porch	81.6	64.7	75.4	0.043
		Near a window	18.4	17.6	18.1	
		Inside the home	11.5	39.2	21.7	
		Other	5.7	3.9	5.1	
	Q4. Among respondents where smoking takes place in the home, near a window or on a porch: Did a neighbor ever complain to you? (N_U_ = 147) Open question: If so, what did you do?	Yes	3.4	13.5	7.1	0.024
		No	96.6	86.5	92.9	
Tobacco Smoke Incursion (TSI)	All respondents: Q5. Did you ever see, fell, or smell tobacco smoke which entered your house as a result of someone smoking in an adjacent apartment, in the hall or in your building? (N_U_ = 284)	Yes	49.3	29.9	44.7	0.009
		No	43.8	55.2	46.5	
		Don't remember	6.9	14.9	8.8	
	Among respondents who ever experienced TSI: Q6. How frequently have you experienced TSI in last month? (N_U_ = 123)	Daily or almost daily	18.3	26.3	19.5	0.132
		Weekly or several times a week	11.5	26.3	13.8	
		1–3 time in a month	17.3	10.5	16.3	
		Didn't penetrate	52.9	36.8	50.4	
	Among respondents who experienced TSI in past month: Q8. To what extent does it disturb you, or not disturb you, when smoke enters your home? (N_U_ = 74)	It disturbed me so much that I sued my neighbors or moved to another apartment	0.0	7.7	1.6	< 0.001
		It disturbs me so much that I am considering suing my neighbors or moving to another apartment	2.0	0.0	1.6	
		It disturbs me a lot	59.2	0.0	46.8	
		It disturbs me	24.5	38.5	27.4	
		It doesn’t disturb me that much	14.3	38.5	19.4	
		It doesn’t disturb me at all	0.0	15.4	3.2	
Risk perceptions	Among respondents who experienced TSI in past month: Q9. To what extent do you think it is harmful, or not harmful, when smoke enters your home? (N_U_ = 74)	Very harmful	36.7	23.1	33.9	0.032
		Pretty harmful	46.9	23.1	41.9	
		Not so harmful	16.3	46.2	22.6	
		Not harmful at all	0.0	1.6	1.6	
	All respondents: Q17. Tobacco smoke is dangerous only if can be smelled (N_U_ = 279)	True	46.0	50.8	47.1	0.501
		Not true	54.0	49.2	52.9	
	All respondents: Q18. Exposure to tobacco smoke on a regular basis: (N_U_ = 279)	Can harm and even kill	77.6	70.8	76.0	0.456
		Can harm but can't kill	21.5	26.2	22.6	
		Can't harm and can't kill	0.9	3.1	1.4	
Responsibility & Rights	All respondents: Q15. Who is primarily responsible for preventing tobacco smoke incursion? (N_U_ = 273)	The government	20.2	4.6	16.5	0.015
		The smoker	54.8	63.1	56.8	
		The resident who suffers from TSI	8.2	10.8	8.8	
		The owner of the smoker's apartment	7.7	3.1	6.6	
		The owner of the apartment into which smoke penetrates	3.4	1.5	2.9	
		Homeowner Association	2.4	4.6	2.9	
		No one needs to prevent this	3.8	12.3	5.9	
	All respondents: Q16. Two neighbors live in apartments, on floors one above the other. The neighbor in the lower floor smokes on his porch, and this very much bothers the neighbor above when smoke enters the apartment With which sentence do you agree more? (N_U_ = 277)	The smoker has a right to smoke anyplace in his own home, even if it bothers neighbors	23.3	54.0	30.2	< 0.001
		It is the right of every person to breath clean air in his/her home, and not to be disturbed by tobacco smoke which penetrates the home	76.7	46.0	69.8	
Support for regulation	All respondents: Q14. Support for smoke-free policy in multi-unit dwellings	Stairway (N_u_ = 283)	80.6	57.6	75.3	< 0.001
		Building entrance (N_u_ = 277)	70.0	32.3	61.2	< 0.001
		Common garden (N_u_ = 280)	50.7	19.7	43.4	< 0.001
		Porch (N_u_ = 281)	40.7	18.2	35.5	< 0.001
		Apartment (N_u_ = 277)	35.0	13.8	30.1	< 0.001
		Support for policy in common areas (N_u_ = 283)	85.3	70.8	81.9	0.008
		Support for policy in private areas (N_u_ = 279)	51.2	25.8	45.2	< 0.001
		Support for any policy (N_u_ = 284)	88.5	74.6	85.2	0.005

(Data weighted for population group, sex, and current smoking status)

The multivariable model showed that type of building was significantly associated with ever experiencing TSI (*p* < 0.001). Living in MUH was associated with 3.6 times the odds of TSI of living in a private home (aOR = 3.60, CI: [1.96–6.58], *p* < 0.001), (Table 3).

**Table 3:** Model results for tobacco smoke incursion, rights of neighbors versus rights of smokers, and support for regulation

*p* value Odds Ratio (OR), 95% Confidence Limits		Ever experienced tobacco smoke incursion 0 = No 1 = Yes (Reference – No) (*N_U_: 247)	Rights of residents of other apartments versus rights of smokers 1 = Rights of smokers 2 – Rights of others (Reference – Rights of Smokers) (*N_U_: 241)	Support for smoke-free legislation of multi-unit dweller buildings (Reference – No) (*N_U_: 247) 0 = No 1 = Yes
Smoking status (Reference: Smokers)	0 = No 1 = Yes	*p* = 0.089 1.83 (0.91,3.68)	*p* = 0.001 3.21 (1.59,6.48)	*p* = 0.011 3.00 (1.28,7.01)
Building type	1 = Multiunit	Overall effect of building: *p* < 0.001	Overall effect of building: * p* = 0.505	Overall effect of building: * p* = 0.437
(1)—Apartment1 versus private home3	2 = Family	*p* < 0.001 3.60 (1.96,6.58)	*p* = 0.280 0.68 (0.34,1.37)	*p* = 0.588 0.79 (0.34,1.84)
(2)—2 family cottages2 versus private home3 (Reference: Private homes)	3 = Private	*p* = 0.443 0.69 (0.27,1.78)	*p* = 0.395 0.65 (0.24,1.76)	*p* = 0.362 1.83 (0.50,6.76)
Educational status (Reference – < 12 years)	0 = < = 12 1 = Above average	*p* = 0.418 1.28 (0.70,2.34)	*p* = 0.639 1.17 (0.61,2.26)	*p* = 0.758 0.88 (0.39,2.00)
Family income (Reference – < average)	0 = Below average/average 1 = Above average	*p* = 0.227 0.68 (0.36,1.27)	*p* = 0.919 1.04 (0.52,2.06)	*p* = 0.163 1.93 (0.77,4.85)
Children under 18 at home (Reference -No)	0 = No 1 = Yes	*p* = 0.906 1.03 (0.59,1.81)	*p* = 0.772 1.09 (0.60,2.00)	*p* = 0.157 0.58 (0.27,1.24)
Sex (Reference – Male)	0 = Male 1 = Female	*p* = 0.623 1.15 (0.66,2.03)	*p* = 0.001 2.77 (1.48,5.16)	*p* = 0.938 0.97 (0.45,2.10)
Age		*p* = 0.913 0.999 (0.98–1.02)	*p* = 0.465 1.01 (0.99,1.03)	*p* = 0.093 0.97 (0.96–1.003)
Ever experienced tobacco smoke incursion (Reference – No)	0 = No 1 = Yes	NR	*p* = 0.414 0.77 (0.40,1.45)	*p* = 0.011 2.99 (1.28–6.97)

(Data weighted for population group, sex, and current smoking status)

*N_U_: **N**umber of **U**nweighted observations

### Risk perceptions and attitudes towards TSI

Among participants who reported TSI in the past month, 77.4% were disturbed by the experience (Smokers: 46.1%, Nonsmokers: 85.7%; *p* value (based on the 6-point ordered scale) < 0.001), and most respondents (75.8%) believed the incursion was harmful (Smokers: 46.2%, Nonsmokers: 83.6%; *p* value (based on the 4-point ordered scale) = 0.032. Nearly half of all respondents (47.1%) thought that tobacco smoke is harmful only if can be smelled, with no difference between smokers and nonsmokers (Smokers: 50.8%, Nonsmokers: 46.0%; *p* = 0.501).

Though 76.0% of respondents thought that regular exposure to SHS can harm or even kill, nearly a quarter of the sample believed that SHS exposure can’t kill even if it occurs regularly (22.6%: Can harm but can’t kill: 22.6%; Can’t harm and can’t kill: 1.4%).

### Responsibility and rights

The most popular response to the question about primary responsibility for preventing TSI was the smoker (56.8%) while 16.5% thought it was the responsibility of the government.

Among all respondents, 69.8% agreed with the statement that an individual has the right to breathe clean air in his/her home, while 30.2% thought it was the right of the smoker to smoke in his/her home even if it bothers others. The multivariable model showed that women and non-smokers were more likely to support the right to breathe smoke-free air (Women versus men: aOR: 2.77 CI [1.48, 5.16], *p* = 0.001; Nonsmokers versus smokers: aOR: 3.21, CI: [1.59, 6.48], *p* = 0.001).

### Support for policy

**Fig. 2:**
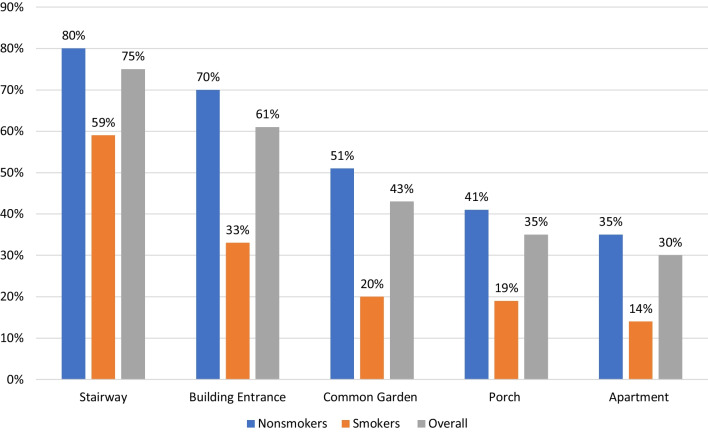
Support for smoke-free legislation in multi-unit dweller buildings

The majority of respondents (85.2%) including three-quarters of smokers, supported some type of legislation for smoke-free MUH, while 81.9% supported smoke-free legislation for common areas, and 45.2% supported smoke-free policies of private spaces. Support was greatest for the public areas of MUH: stairways 75.3%, building entrances 61.2%, common gardens 43.3%, but lower for private areas: porches 35.5%, and other indoor areas: 30.1% (See Fig. 2).

The multi-variable model showed that those ever-exposed to TSI and non-smokers were more likely to support legislation (ever-exposed versus never-exposed aOR = 2.99, CI: [1.28, 6.97], *p* = 0.011; nonsmokers versus smokers aOR = 3.00, CI [1.28, 7.01], *p* = 0.011).

## Discussion

Close to half of respondents in this study reported exposure to TSI. Among those reporting incursion in the past month, 77.4% were troubled by it, and 75.8% believed it to be harmful. While there was a broad consensus (69.8% of respondents, including nearly half of current smokers) that favored a resident’s right to breathe smoke-free air over the right of a smoker to smoke anywhere in their apartment, only about a quarter of affected respondents challenged their neighbors, neighbors' landlords, or building committees to change the neighbor’s smoking behavior. Most of those who did not speak up felt it was not their right to tell someone to refrain from smoking in their own apartment, were afraid of conflict with the neighbor, or believed it wouldn't help. A large majority of respondents favored legislation and policy to limit exposure to tobacco smoke incursion in multi-unit apartment buildings, with support highest for stairways and building entrances. A greater proportion of non-smokers supported protection policies relative to smokers.

Our findings compare quite closely with recent international studies, which suggest that TSI is a common experience among non-smokers living in multi-unit dwellings. We found that 49.3% of non-smokers reported ever experiencing TSI in their home, with 33.3% of those respondents reporting TSI occurring weekly or more frequently. Studies from the large urban setting in the United States report TSI exposure among non-smokers ranging from 22.9 to 33.5% in the past year [25–28]. Other countries provide a range of estimates, including a high rate of 74.7% of non-smokers reporting TSI in the past year in Korea [29], to 6.1 % in Poland in the past month [30]. Notably, international studies reported high rates of support for a total smoking ban in all areas of multiunit dwelling properties. An earlier survey from the U.S. found that 91.3% of residents living in multi-unit dwellings believe that residents have a right to live in a tobacco smoke-free building [28], reflecting the strong support for comprehensive smoke-free rules in the present study. Even a substantial minority of smokers support far-reaching rules: 36.1% of smokers in the U.S., Canada and the U.K. reported a preference for a property-wide ban on smoking.

In liberal democracies such as Israel, there is a reluctance to regulate personal behavior unless it harms others: personal practices in private spaces are even more difficult to legislate. Consistent with this approach, smoking bans in private spaces have received far less attention than bans on smoking in public places. Yet, the World Health Organization defines clean air as a "basic human right" [31]. The health damage from secondhand smoke exposure is well-documented and has led to restrictions on smoking in countries around the world [1, 2]. Research into damage from TSI is more recent and less prolific, but, as reported above, TSI may increase illness among young children and adolescents [14, 15]. Further, there is good scientific evidence that smoke from outside can contaminate smoke-free indoor areas, through the mechanism known as smoke drift [32–34], and that children living in non-smoking apartments in multi-unit dwellings have higher levels of exposure to tobacco smoke, as evidenced by biomarkers, than children in non-smoking apartments in private dwellings [13]. The large decreases in hospitalizations for acute coronary syndrome in adults [35] and child hospitalizations for asthma attacks [36] subsequent to passage of smoke-free laws suggests that such decreases in adverse outcomes and improvements in the health of the public are likely to occur in Israel if TSI is restricted.

This provides justification for regulations that would protect the public from TSI in private homes, which have become the primary place of exposure for adults and children alike [8]. To date, there have been few regulations imposed on smoking in private homes, and protections have been gained mostly through voluntary adoption of smoking bans by private property owners or landlords [37]. A federal rule instituted in the U.S. in 2018 banned indoor smoking in all public housing buildings, including the private homes of some 2 million residents [38, 39]. Other examples include smoke-free rules for foster homes, and court-imposed requirements regarding smoking near children. For example, a California trial court judge ordered a mother not to smoke in the presence of her child within the home [9]. A limited set of local jurisdictions have adopted smoke-free home ordinances in the U.S. [40] and Australia [8, 10], and the Australian state of New South Wales has ruled that people are protected from smoke-drift in certain types of apartments [41].

**Fig. 3:**
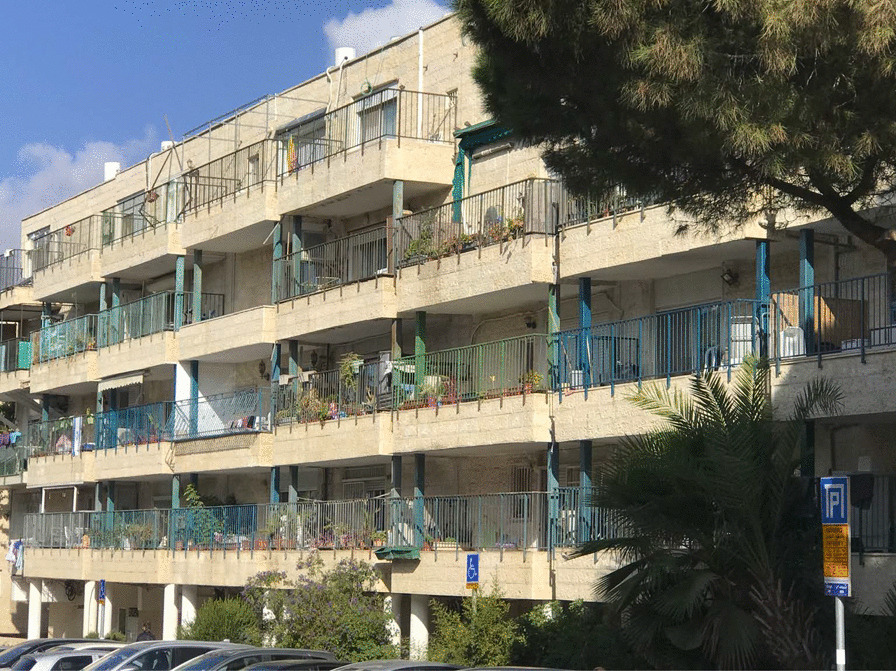
Photograph of multi-unit apartment building in Jerusalem

The problem is compounded among the most vulnerable members of the population. Persons of lower socio-economic status are concentrated more heavily in smaller dwellings, and have both higher smoking rates and higher rates of exposure to tobacco smoke [42]. The minimum distance of 9 meters in outdoor areas required to protect most people from exposure to emissions from smoking a single cigarette [43] will often be unmet in Israeli apartments, particularly among those of lower socioeconomic status. Figure 3 shows an apartment building in Jerusalem. In that setting, the smoke emissions from one person smoking a single cigarette on a porch can reach at least 10 neighboring apartments.

While indoor smoking bans in public places have engendered some controversy, the proposal to impose a limit on smoking that results in home-based incursion of neighboring residences represents a new and challenging approach to protecting the public’s health. The present findings suggest that not only are interventions needed, but some would be considered appropriate by a majority of Israelis. Nonetheless, any regulation that imposes limits on the personal behavior of people in their own homes is likely to face substantial obstacles. The first obstacle is the difficulty in enforcing a ban on smoking in proximity to others, because domestic environments are not typically or easily visible to traditional law enforcement agencies. The challenge of ensuring effective enforcement may also constitute a deterrent to the enactment of such legislation in the first place. An unenforceable policy may produce the unintended consequence of legitimizing non-compliance. Complaint-based enforcement, without objective data indicating non-compliance with the smoking ban, is equally problematic due to its potential for abuse. At the same time, the requirement to present objective evidence (such as air quality testing) will make enforcement complex for the injured party and possibly invasive for smokers. This is especially problematic because the burden of SHS exposure is concentrated on lower income communities [12, 44–46].

Despite the complexities of enforcement of legislation, policy action will likely raise consciousness and help change social norms about the issue. Further, a “feedback loop” has been identified, which could benefit the population: implementation of smoke-free policies has been shown to lead to increased support for those policies [47, 48].

The results of our study show broad support for bans on smoking in stairways (75%) and in building entrances (61%). As such, it provides support for initiating legislation regarding these areas immediately. Support for blanket bans on smoking on porches (35%) and in indoor areas of apartments (30%) was much lower. However, the finding that a large majority—70% of respondents—favored the rights of people to breathe smoke-free air in their own homes suggest that there is support for protection from TSI *when it occurs*. This is the argument being used currently in the Supreme Court case 1416/21: the plaintiffs are not asking for a blanket ban on smoking inside of apartments or porches, but are requesting that existing laws be interpreted to prevent TSI in the event that it occurs.

In tobacco control, the path to protection of the public sometimes begins not in the legislature but in the courts. This was true, for example, regarding smoke-free air travel: a court case brought to the Israeli Supreme Court against the Ministry of Transportation resulted in bans on smoking in all flights entering and leaving Israel beginning in 1998, and the case brought in a Miami Court against the tobacco industry for harm caused to flight attendants contributed to bans on smoking in all areas controlled by the U.S. Federal Aviation Authority. Many other countries followed suit [49, 50]. In 2018, a bill to prevent smoking on porches was blocked in the governmental committee on legislation, and failed to reach the first reading in the Israeli Knesset [51]. A recent court case regarding tobacco smoke incursion resulted in compensation from the smokers to the neighbors who brought the case [52]. In 2021, an administrative case (Bagatz 1416/21) against the Ministers of the Environment, Health, and Public Security (Police) was brought to the Supreme Court by the non-governmental organization Avir Naki (Clean Air Society) and 6 individuals who believed that they had suffered due to TSI. One non-smoker had had a severe heart attack, and his doctors (incorrectly) thought he was a heavy smoker based on hospital testing; he was in fact a lifelong nonsmoker, but heavily exposed due to TSI into his own apartment. Another became ill with cancer, and another suffered from breathing difficulties and allergies, and was unable to sleep inside of her apartment. The petitioners requested that TSI be recognized as a hazard, an odor nuisance and air pollution under existing Israeli laws. The request was for a **partial** ban, which would be applicable when tobacco smoke creates a nuisance under the law. This differs from the USA solution of a total ban on smoking anyplace inside or on the grounds of federal public housing throughout the country. The case was heard for the first time in 2022. As of the time of this writing, no decision has been reached [53].

### Strengths and limitations

To the best of our knowledge, this is the first study to assess TSI into Israeli homes. It is also the first study of which we are aware which specifically addressed rights of smokers to smoke in their homes versus rights of neighbors to breathe smoke-free air in their own homes. To achieve a nationally representative sample of cellphone owners in Israel, a random digit dial survey was adopted, with data weighted for population group, sex, and smoking status. We were unable to weight the data on additional variables due to the size of the study. Potential biases regarding willingness of respondents to participate in a survey may pose a threat to generalizability. The survey was run in Hebrew only, so those who did not speak Hebrew well enough to answer were excluded. According to the Central Bureau of Statistics, 8% of Arab men and 25% of Arab women speak little to no Hebrew [54]. While the relatively small sample size was sufficient to address the primary objectives of the study, our ability to detect small effects in the multivariable analyses was limited, and estimates of percentages in some cells may be based on small numbers. These data are cross-sectional, and all relationships are associations. TSI was based on self-report. Further, though we asked about support for legislation of various areas in and around multi-unit dwellings, we did not ask directly about support for legislation which would protect people from experiencing TSI when it occurs, without imposing a blanket ban. Respondents may not have had complete knowledge of the source of TSI. We classified those who responded that they smoked at social events, reserve military duty, or in periods of stress as nonsmokers. Some investigators may have categorized these individuals as smokers, while others would have included them as nonsmokers. We do not have any information on those who didn’t respond at all; these individuals may differ from those who did respond. The data were collected in 2017, and it is possible that some changes may have occurred since then. First, changes in TSI may have occurred as a result of the broad societal changes which occurred during COVID-19. A study done in Israel following the first COVID-19 lockdown showed that while smoking behavior was mostly unchanged in the population following the first lockdown, the proportion of people smoking in the home may have increased [23]. Because of the physical proximity of neighboring apartment dwellings, increased home smoking may have led to increased levels of TSI. Second, recent high-profile court cases about TSI and the widely discussed administrative case which is currently being heard in the Supreme Court may have led to changes in TSI. Nonetheless, to the best of our knowledge, this is the first formal investigation of TSI in Israel. We are not aware of other studies that have assessed public perceptions of the rights of smokers compared with the rights of nonsmokers in the context of neighbor smoking. Further study, which is more recent and is based on a larger sample size, is important for enhancing the evidence base for policy, media, and other interventions.

## Conclusions

Among study participants, tobacco smoke incursion was a common, yet unwelcome experience. Overwhelmingly, participants supported the right of neighbors to breathe clean air over the right of smokers to smoke in a manner that results in smoke incursion in neighbors’ homes, yet most did not confront their neighbors.

The problem of TSI is particularly salient globally in areas of high smoking rates and high population density such as Israel. Moreover, this problem both in Israel and elsewhere may not remain focused on tobacco smoke: the liberalization of cannabis use regulations in a number of international jurisdictions may lead to other forms of smoke incursion problems. Policy action could be strengthened by further research on TSI, including prevalence of exposure and its correlates, associated health risks, and public preferences regarding protection from TSI globally.

Nevertheless, the existing evidence both in Israel and from elsewhere suggests that decisive governmental intervention and policies to change social norms are needed to ensure that the public is protected from persistent exposure to tobacco smoke incursion in their own homes. In Israel, legal action at the level of the Supreme Court or the Knesset, as well as policy initiatives and campaigns to denormalize smoking in proximity to other people, will likely reduce the scope of this widespread problem, protect individuals from home TSI, and improve population health.

## Data Availability

Data analysed in the current study are available from the corresponding author on reasonable request.

## Abbreviations

TSI: Tobacco smoke incursion (TSI)
SHS: Secondhand smoke
MUH: Multi-unit housing
aOR: Adjusted odds ratio
CI: Confidence interval
WHO: World Health Organization
FCTC: Framework convention for tobacco control
CBS: Central Bureau of Statistics
AAPOR: American Association of Political Opinion Research
